# Pilot Study: Long-Term Shedding of SARS-CoV-2 in Urine: A Threat for Dispersal in Wastewater

**DOI:** 10.3389/fpubh.2020.569209

**Published:** 2020-11-23

**Authors:** Andreina Baj, Lorenzo Azzi, Daniela Dalla Gasperina, Angelo Genoni, Antonio Tamborini, Cinzia Gambarini, Giulio Carcano, Paolo Grossi, Fausto Sessa

**Affiliations:** ^1^Laboratory of Clinical Microbiology, Azienda Socio Sanitaria Territoriale (ASST) Sette Laghi, Department of Medicine and Surgery, University of Insubria, Varese, Italy; ^2^Unit of Oral Medicine and Pathology, Azienda Socio Sanitaria Territoriale (ASST) Sette Laghi, Department of Medicine and Surgery, University of Insubria, Varese, Italy; ^3^Unit of Infectious and Tropical Diseases, Azienda Socio Sanitaria Territoriale (ASST) Sette Laghi, Department of Medicine and Surgery, University of Insubria, Varese, Italy; ^4^S.C. Pneumology, Azienda Socio Sanitaria Territoriale (ASST) Sette Laghi, Ospedale di Circolo e Fondazione Macchi, Varese, Italy; ^5^Unit of General Emergency and Transplant Surgery, Azienda Socio Sanitaria Territoriale (ASST) dei Sette Laghi, Department of Medicine and Surgery, University of Insubria, Varese, Italy; ^6^Pathology Unit, Azienda Socio Sanitaria Territoriale (ASST) Sette Laghi, Department of Medicine and Surgery, University of Insubria, Varese, Italy

**Keywords:** shedding, wastewater, urine, COVID-19, SARS CoV-2

## Abstract

Only 4 months after the beginning of SARS-CoV-2 epidemic, the world is facing a global pandemic due to a complex and insidious virus that today constantly poses new challenges. In this study, we highlight a persistent shedding of SARS-CoV-2 RNA into the urine, even in patients with a negative nasopharyngeal swab and in patients considered recovered. What does it mean? Besides the fact that the kidney is a probable site of viral replication, the prolonged viral excretion is a matter of great concern for our drainage system contamination.

## Introduction

After having looked at China as the place of onset of an epidemic in December 2019, on February 18, 2020 we recorded the first case of COronaVIrus Disease 2019 (COVID-19) in Italy. Since then, in only 2 months, our reports have accounted for 233,000 cases and more than 33,000 deaths (1).

The viral agent, SARS-CoV-2, belongs to *Coronaviridae* family and, together with SARS-CoV and MERS-CoV, is the third zoonotic coronavirus that, in recent history, has emerged in the human population causing severe and unexpected disease outbreaks (2, 3).

In this storm that has caught everyone unprepared, we have tried to cope with many issues related to the diagnosis and management of this disease, and now many others are coming to light. The gold standard to detect the virus in the patients affected by COVID-19 is an rRT-PCR on their nasopharyngeal swabs, both for diagnosis and follow-up. After two negative swabs, patients are discharged but the SARS-CoV-2 RNA could be still present in the saliva (4, 5), and probably in other body districts, possibly acting as a viral reservoir (6).

In this study, we demonstrate that SARS-CoV-2 RNA is detected in urine of COVID-19 patients even when the patient is no longer symptomatic and in some cases for a long time.

From this perspective, a prolonged viral shedding into the urine could be a serious environmental problem because the virus enters the drainage system of urban and non-urban centers, making its presence everlasting (7). Taking into account that, according to the United Nations, more than 80% of the world's wastewater flows back into the environment without treatment (8), it is not difficult to imagine sewage as a viral reservoir that continuously contaminates the environment.

Of interest, even if fecal shedding of SARS-CoV-2 RNA is frequently observed in COVID-19 patients and it supports a productive infection of enterocytes, it is rapidly inactivated after its release into the intestinal lumen (9). This observation raises the need for further studies to evaluate the viability of SARS-CoV-2 in different environments to assess its infectivity.

## Materials and Methods

### Patient Recruitment

Twenty-two SARS-CoV-2–infected patients with severe or very severe disease were recruited into the study following the consecutive sampling technique, in agreement with the Helsinki declaration. Patients were admitted to our hospital (ASST Sette Laghi, Lombardia, Italy) after being diagnosed with COVID-19 through an rRT-PCR on their nasopharyngeal swabs (GeneFinder COVID-19 Plus Real *Amp* Kit; Elitech, Puteaux, France). Their clinical situation was classified according to the Diagnosis and Treatment Plan of COVID-19 issued by the Chinese National Health Commission (10).

A single urine sample was obtained from each patient, in conjunction with the second nasopharyngeal swab.

### Nucleic Acid Extraction and rRT-PCR

One milliliter of urine specimens was centrifuged at 1,500 rpm for 5 min; the obtained pellets, resuspended in 140 μl of phosphate buffered saline, were subjected to RNA extraction by QIAmp Viral RNA mini kit (Qiagen) according to the manufacturer's instructions and eluted in 60 μl. One-step rRT-PCR was performed using Luna Universal qPCR Master Mix (New England Biolabs) from 5 μl of extracted RNA and a SYBR GREEN detection method. Forward (5′-ACCTTCCCAGGTAACAAACCA-3′) and reverse (5′-TTACCTTTCGGTCACACCCG-3′) primers targeting the 5′UTR region of SARS-CoV-2, designed using CLC genomics workbench (QIAGEN), were used (4, 5).

**Figure 1:**
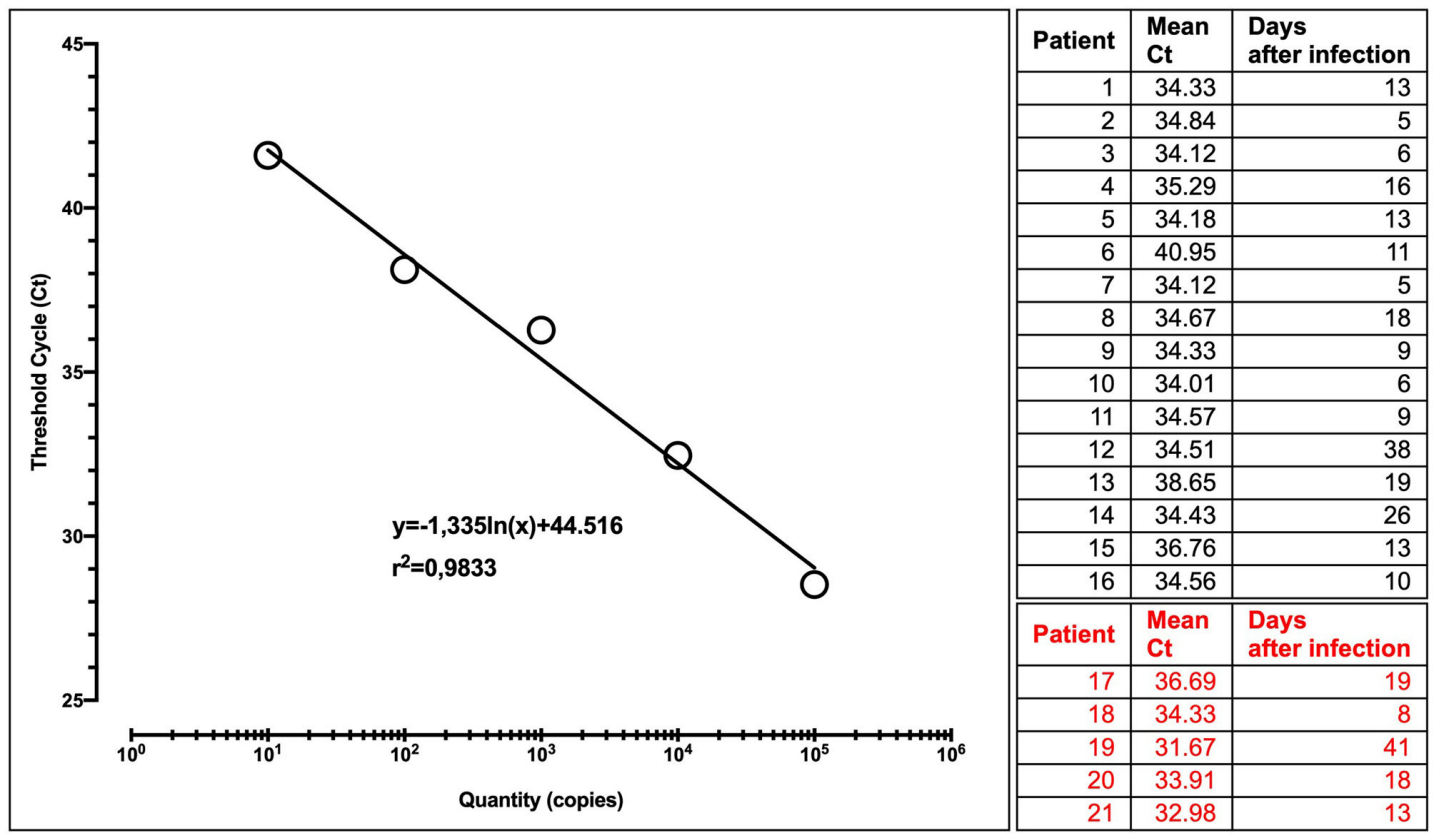
Calibration curve with the regression equation (characterized by the slope and intercept) and regression coefficient. Patients tested at different times after infection are shown. In red, patients with a precedent or contextually negative nasopharyngeal swab.

All samples were run in four replicates, together with urine from healthy people as a negative control, and a quantified positive control (Twist Bioscience SARS-CoV-2 RNA Control). Provided at a concentration of 10^6^ copies μl, serial dilutions of RNA control from 10^5^ to 10 copies/μl were used to construct a standard curve (Figure 1).

RT-PCR was performed using Abi Prism 7000 sequence detection system (Applied Biosystems), with an annealing temperature of 60°C.

In case of uncertain result, an endpoint RT-PCR and subsequent sequencing of 5′UTR region was performed on the same RNA. To this end, the 251-bp amplicon was detected with LabChipGx Touch24 (Perkin Elmer) and sequenced by the Sanger method (SeqStudio Genetic Analyzer; Thermo Fisher Scientific).

## Results

A total of 22 subjects, 14 males and 6 females, were analyzed in this study. Age values ranged from 37 to 82 years, with a mean age of 61 ± 12.5 years.

All patients were affected by severe or very severe COVID-19 and hospitalized in the Intensive Care Unit, in the Unit of Infectious and Tropical Diseases, and in Pneumology.

In all observed patients, microhematuria was present, associated with proteinuria in 70% and hypoalbuminemia in 89% of the subjects. Serum creatinine and urea level were higher than normal in 45 and 62% of the patients, respectively (data not shown).

Interestingly, in all patients but one (95%), we found RNA of SARS-CoV-2 in urine sediment, in a period lasting until 41 days after the onset of COVID-19 symptoms (Figure 1).

Of note, in five patients (23%), viral RNA was present in urine but no longer detectable in nasopharyngeal swab at the time of enrollment in the study. Among them, a patient with a constantly negative nasopharyngeal swab for 30 days but with a high urine viral load after 41 day after the onset of symptoms is of particular interest.

## Discussion

With time, many aspects of SARS-CoV-2 infection are coming to light. In these last few months, scientific evidence has shown us that it is a respiratory virus, but the resulting infection is not localized, and clinical pictures are suggestive of extrapulmonary viral dissemination. Furthermore, virus shedding could occur from different sites and not only through respiratory droplets (6, 7).

In our study, we demonstrated a significant presence of viral genome in the urine of COVID-19 patients, even when the virus RNA itself was no longer detectable at nasopharyngeal level and also when symptoms had disappeared.

This paper is part of an ongoing study and the number of patients analyzed at this moment does not allow to establish a correlation between renal damage and viral presence in urine. Nevertheless, the presence of virus receptor ACE2 on podocytes and tubular cells was described; thus, we could presume a possible direct infection of the kidney (11) and additional studies reported the kidney as specific target for SARS-CoV-2 infection (12). In a recent study, acute kidney injury developed in 36.6% of the patients admitted with COVID-19, with high rates of proteinuria and hematuria (13).

With this preliminary work, we would like to draw close attention to the growing concern for the environment, especially in some cities and areas with a high population density. A prolonged shedding of the virus into the urine, even when the patient is no longer symptomatic and with negative nasopharyngeal swabs, could mean a virus release into the sewage system and, through it, its spread in the environment.

Many aspects of SARS-CoV-2 remain to be considered: its viability in different body fluids, its environmental stability, and its transmission via different routes, but this finding raises a concern about the management of recovering patients and, above all, the treatment of wastewater. Strict monitoring of this process is essential to prevent viral contamination. Further studies are needed to investigate these hypotheses.

## Data Availability Statement

The raw data supporting the conclusions of this article will be made available by the authors, without undue reservation.

## Ethics Statement

The studies involving human participants were reviewed and approved by This study was carried out in agreement with the Helsinki declaration, authorized by the Hospital Direction, due to the situation of emergency and approved by the Ethical Committee of the University of Insubria (study n° 68/2000). The patients/participants provided their written informed consent to participate in this study.

## Author Contributions

AB, LA, DD, and FS contributed to study design, data collection, data interpretation, the literature search, and writing of the report. CG, GC, and PG contributed to patients' recruitment, data collection, and clinical management. AT managed the laboratory diagnostic procedures and collected data. AG managed the laboratory experimental procedures. All authors contributed to the article and approved the submitted version.

## Conflict of Interest

The authors declare that the research was conducted in the absence of any commercial or financial relationships that could be construed as a potential conflict of interest.
